# Postoperative Aspiration Pneumonia Among Adults Using GLP-1 Receptor Agonists

**DOI:** 10.1001/jamanetworkopen.2025.0081

**Published:** 2025-03-04

**Authors:** Yuan-Hsin Chen, Thomas Zink, Ya-Wen Chen, Darren Z. Nin, Carl T. Talmo, Brian L. Hollenbeck, Andrew R. Grant, Ruijia Niu, David C. Chang, Eric L. Smith

**Affiliations:** 1 Department of Surgery, Massachusetts General Hospital, Harvard Medical School, Boston; 2 Codman Center for Clinical Effectiveness in Surgery, Massachusetts General Hospital, Boston; 3 Department of Orthopedic Surgery, New England Baptist Hospital, Boston, Massachusetts; 4 Department of Orthopedic Surgery, Tufts Medical Center, Boston, Massachusetts

## Abstract

**Importance:**

Glucagon-like peptide-1 receptor agonists (GLP-1 RAs) have been increasingly prescribed for weight management in recent years. However, little is known about whether preoperative GLP-1 RA use is associated with a greater risk of complications after surgery.

**Objective:**

To investigate whether preoperative GLP-1 RA use is associated with postoperative aspiration pneumonia among patients undergoing common surgical procedures.

**Design, Setting, and Participants:**

This retrospective cohort study, conducted using MarketScan commercial claims databases, included patients who underwent 1 of 14 common surgical procedures from April 1, 2020, to September 30, 2022. Individuals who were younger than 18 years, underwent multiple surgical procedures, or had a preoperative history of pneumonia or acute respiratory failure in the 90 days preceding surgery were excluded. Data were analyzed from December 2023 to March 2024.

**Main Outcome and Measures:**

The primary outcome was aspiration pneumonia in the 30-day postoperative period. Multivariable logistic regressions were performed, adjusting for patient and surgical characteristics.

**Results:**

This study included 366 476 patients (median age, 53 years [IQR, 43-62 years]), of whom 56.4% were women. In the cohort, 5931 patients (1.6%) had a preoperative prescription for a GLP-1 RA. Patients using GLP-1 RAs were more likely to be female (3502 [59.0%] vs 203 288 [56.4%]) and diagnosed with both obesity and diabetes (2819 [47.5%] vs 24 635 [6.8%]) compared with nonusers. Adjusted analysis showed no significant differences in the odds of postoperative pneumonia (odds ratio, 0.78; 95% CI, 0.57-1.06; *P* = .12) between GLP-1 RA users and nonusers.

**Conclusions and Relevance:**

This cohort study found no significant association between the preoperative use of GLP-1 RAs and short-term postoperative aspiration pneumonia despite growing concerns about the adverse effects of these medications after surgery. This finding suggests that it may be beneficial to reassess the preoperative withholding guidelines for GLP-1 RAs.

## Key Points

**Question:**

Is preoperative use of glucagon-like peptide-1 receptor agonists (GLP-1 RAs) associated with respiratory surgical complications?

**Findings:**

In this cohort study including 366 476 individuals undergoing common surgical procedures, patients taking GLP-1 RAs had no significant differences in odds of postoperative aspiration pneumonia compared with patients not using GLP-1 RAs after adjusting for patient demographics, comorbidities, and surgical characteristics.

**Meaning:**

In this study, prevalence of postoperative aspiration pneumonia was not higher among patients using GLP-1 RAs, suggesting that it might be beneficial to reassess the preoperative withholding guidelines for these medications.

## Teaser

This cohort study investigates whether use of glucagon-like peptide-1 receptor agonists (GLP-1 RAs) is associated with postoperative aspiration pneumonia among patients undergoing common surgical procedures.

## Introduction

Use of glucagon-like peptide-1 receptor agonists (GLP-1 RAs) is rapidly increasing following their approval for weight management due to their effectiveness in weight reduction and their accessibility compared with bariatric surgery.^1,2,3^ However, as the popularity of GLP-1 RAs has grown, concerns regarding their adverse effects and perioperative safety have surfaced.^4,5,6^ Notably, the risk of aspiration under anesthesia for GLP-1 RA users has raised considerable attention.^7,8,9^ The basis for this concern is that GLP-1 RAs are associated with delayed gastric emptying, resulting in increased residual gastric content and adverse gastrointestinal events, such as vomiting and gastroesophageal reflux.^6,10^ Aspiration of gastric contents carries the potential for severe complications, including life-threatening pneumonia.^11^ Therefore, in 2023, the American Society of Anesthesiologists (ASA) recommended discontinuing GLP-1 RA use for up to a week before surgery.^12^

The association between GLP-1 RA use and postoperative aspiration pneumonia remains inconclusive. While some studies reported higher risk in patients using GLP-1 RAs, others found no significant difference.^8,9,13,14,15,16,17,18,19^ It is imperative to ensure the safety of surgical patients who use GLP-1 RAs, especially considering the increasing number of people using these drugs. Therefore, this study aimed to elucidate the association between GLP-1 RA use and aspiration pneumonia in the period before the 2023 ASA recommendation.

## Methods

### Data Source

This cohort study used data from the MarketScan database, which includes over 273 million unique patients annually, comprising insured employees, retirees, and their spouses and dependents covered by employer-sponsored private health insurance in the US.^20^ This comprehensive dataset enables longitudinal tracking of patients across various institutions and practitioners, making it highly suitable for studying health care utilization and outcomes. It includes detailed information on outpatient prescription drug use, inpatient and outpatient medical procedures, diagnoses, and services. Specifically, the Commercial Claims and Encounters Database offers detailed medical and pharmaceutical data, while the Inpatient Services and Outpatient Claims files contain claims with actual dates of service for professional encounters and services.^21^ This study was approved by the New England Baptist Hospital institutional review board and followed the Strengthening the Reporting of Observational Studies in Epidemiology (STROBE) reporting guideline.^22^ Informed consent was waived because the research presented no more than minimal risk of harm to participants and involved no procedure for which written consent is normally required outside the research context.

### Inclusion and Exclusion Criteria

We conducted a retrospective cohort study that included patients who underwent 1 of 14 common, high-volume surgeries in the US between April 1, 2020, and September 30, 2022. These surgical procedures were chosen as exemplars of frequently performed surgeries, providing a broad yet representative sample.^23^ The procedures included groin hernia repair, hysterectomy, knee arthroplasty, hip arthroplasty, appendectomy, laminectomy, bariatric surgery, spinal fusion, tibial and ankle shaft fractures, colectomy, lower extremity amputation, thyroidectomy, hip fracture, and coronary artery bypass graft.^23^ All surgeries were identified using *Current Procedural Terminology* codes (eTable 1 in Supplement 1).

We excluded individuals who were younger than 18 years, underwent multiple surgeries, or had a 90-day preoperative history of pneumonia or acute respiratory failure. Additionally, patients without continuous enrollment in a health insurance plan in the MarketScan dataset for 90 days before and 90 days after their surgery date were excluded (eFigure in Supplement 1).

### Primary Variables and Covariates

The primary independent variable was the use of a GLP-1 RA, which was defined as having any prescription record of dulaglutide, exenatide, liraglutide, or semaglutide within 30 days prior to the index surgery. Given the current uncertainty regarding the duration of delayed gastric emptying induced by GLP-1 RAs, with some studies reporting delays lasting up to 16 weeks,^18,19,24,25,26^ there is no universally agreed-upon cutoff for the exposure period. However, considering the potential for ongoing gastric delay during surgery, it is reasonable to define exposure as 30 days before surgery.

The primary outcome was 30-day postoperative aspiration pneumonia, identified using *International Statistical Classification of Diseases and Related Health Problems, 10th Revision (ICD-10)* codes (eTable 2 in Supplement 1). Given the uncertainty regarding the duration of delayed gastric emptying caused by GLP-1 RAs, we adopted a 30-day follow-up period to ensure comprehensive postoperative monitoring. The secondary outcome was 90-day postoperative acute respiratory failure, a common respiratory complication following surgery, identified using *ICD-10* codes (eTable 2 in Supplement 1).

Covariates were patient demographics, comorbidities, and surgical acuity. Patient demographics included age, sex, and geographic region (Northeast, Midwest, South, or West). Patient comorbidities were identified based on *ICD-10* codes in diagnosis records within 90 days prior to the date of surgery. These included diabetes, obesity, hypertension, active cancer, chronic obstructive pulmonary disease, chronic kidney disease, dementia, gastroesophageal reflux disease, dysphagia, gastroparesis, myocardial infarction, heart failure, peripheral vascular disease, cerebrovascular disease, connective tissue disease, peptic ulcer disease, liver disease, hemiplegia, and AIDS.

We further grouped the surgeries into 3 categories of acuity based on how often they are done electively vs urgently at the population level: (1) regularly elective (bariatric surgery, knee arthroplasty, hip arthroplasty, and colectomy), (2) mixed elective and urgent (groin hernia repair, hysterectomy, laminectomy, spinal fusion, lower extremity amputation, thyroidectomy, and coronary artery bypass graft), and (3) regularly urgent or emergent (appendectomy, hip fracture, and tibial and ankle shaft fractures).^23^

### Statistical Analysis

We assessed differences in characteristics between patients with and without GLP-1 RA prescriptions using standardized mean differences (SMDs), with an SMD less than 0.1 indicating adequate balance between groups. Multivariable logistic regressions were used to adjust for patient demographics (age, sex, and geographic region), aforementioned comorbidities, and surgical acuity.

Sensitivity analyses were performed to ensure robustness of the findings. These included (1) re-estimating the model by adjusting for surgical heterogeneity with indicator variables for each specific type of surgery, (2) re-estimating the model by incorporating the anesthesia type that is generally done for selected surgeries at the population level, (3) restricting the sample to patients with indications of GLP-1 RA use (ie, diabetes or obesity diagnoses), (4) excluding patients who died during the index admission, and (5) re-estimating the model using propensity score analysis. All effect estimates are reported with corresponding 95% CIs, and statistical significance was defined as 2-sided *P* < .05. All analyses were performed from December 2023 to March 2024 using Stata, version 17.0 (StataCorp LLC).

## Results

### Baseline Population Demographics

A total of 366 476 patients were included in the study, with a median age of 53 years (IQR, 43-62 years); 43.6% were men and 56.4% were women. Among them, 5931 (1.6%) used GLP-1 RAs. Most patients received semaglutide (2543 [42.9%]) followed by dulaglutide (2293 [38.7%]), liraglutide (945 [15.9%]), and exenatide (161 [2.7%]). The 3 most common surgeries were groin hernia repair, hysterectomy, and knee arthroplasty (eTable 3 in Supplement 1).

### Unadjusted Analysis

Patients using GLP-1 RAs compared with not using GLP-1 RAs were more likely to be female (3502 [59.0%] vs 203 288 [56.4%]), to be aged 46 to 62 years (3629 [61.2%] vs 171 063 [47.4%]), to live in the South (3077 [51.9%] vs 162 278 [45.1%]), and to have been diagnosed with both obesity and diabetes (2819 [47.5%] vs 24 635 [6.8%]), hypertension (4127 [69.6%] vs 146 736 [40.7%]), or chronic kidney disease (378 [6.4%] vs 10 436 [2.9%]) (Table 1). Additionally, a higher proportion of patients using GLP-1 RAs developed acute respiratory failure compared with patients not using GLP-1 RAs (799 [13.5%] vs 40 060 [11.1%]; *P* < .001). In contrast, the proportion with postoperative aspiration pneumonia was similar in both groups (42 [0.7%] vs 2335 [0.6%]; *P* = .56) (Table 2).

**Table 1.** Characteristics of Patients by Use of GLP-1 RAs

Characteristic	Patients, No. (%) (N = 366 476)		SMD (95% CI)^a^
	GLP-1 RA users (n = 5931)	GLP-1 RA nonusers (n = 360 545)	
Sex			
Female	3502 (59.0)	203 288 (56.4)	0.05 (0.03 to 0.08)
Male	2429 (41.0)	157 257 (43.6)	
Age group, y			
<46	1062 (17.9)	111 363 (30.9)	0.31 (0.28 to 0.33)
46-55	1840 (31.0)	89 631 (24.9)	0.14 (0.11 to 0.16)
56-62	1789 (30.2)	81 432 (22.6)	0.17 (0.15 to 0.20)
≥63	1240 (20.9)	78 119 (21.7)	0.02 (−0.01 to 0.04)
Comorbidities			
No diabetes or obesity	342 (5.8)	212 506 (58.9)	1.38 (1.36 to 1.41)
Obesity only	592 (10.0)	96 428 (26.7)	0.44 (0.42 to 0.47)
Diabetes only	2178 (36.7)	26 976 (7.5)	0.75 (0.73 to 0.78)
Diabetes and obesity	2819 (47.5)	24 635 (6.8)	1.03 (1.00 to 1.05)
Hypertension	4127 (69.6)	146 736 (40.7)	0.61 (0.58 to 0.63)
Cancer	526 (8.9)	31 021 (8.6)	0.01 (−0.02 to 0.04)
COPD	290 (4.9)	18 989 (5.3)	0.02 (−0.01 to 0.04)
Chronic kidney disease	378 (6.4)	10 436 (2.9)	0.17 (0.14 to 0.19)
Dementia	15 (0.3)	1846 (0.5)	0.04 (0.02 to 0.07)
GERD	926 (15.6)	53 480 (14.8)	0.02 (0.00 to 0.05)
Dysphagia	83 (1.4)	3705 (1.0)	0.03 (0.01 to 0.06)
Gastroparesis	31 (0.5)	503 (0.1)	0.07 (0.04 to 0.09)
Myocardial infarction	147 (2.5)	5705 (1.6)	0.06 (0.04 to 0.09)
Heart failure	268 (4.5)	8242 (2.3)	0.12 (0.10 to 0.15)
Peripheral vascular disease	306 (5.2)	10 251 (2.8)	0.12 (0.09 to 0.14)
Cerebrovascular disease	191 (3.2)	7296 (2.0)	0.07 (0.05 to 0.10)
Connective tissue disease	109 (1.8)	6851 (1.9)	<0.01 (−0.02 to 0.03)
Peptic ulcer disease	23 (0.4)	1492 (0.4)	<0.01 (−0.02 to 0.03)
Liver disease	392 (6.6)	15 755 (4.4)	0.10 (0.07 to 0.12)
Hemiplegia	23 (0.4)	1473 (0.4)	<0.01 (−0.02 to 0.03)
AIDS	19 (0.3)	834 (0.2)	0.02 (−0.01 to 0.04)
Surgical procedure			
Bariatric surgery	603 (10.2)	20 535 (5.7)	0.17 (0.14 to 0.19)
Knee arthroplasty	1128 (19.0)	52 713 (14.6)	0.12 (0.09 to 0.14)
Hip arthroplasty	471 (7.9)	33 647 (9.3)	0.05 (0.02 to 0.08)
Colectomy	139 (2.3)	12 469 (3.5)	0.07 (0.04 to 0.09)
Laminectomy	444 (7.5)	25 348 (7.0)	0.02 (−0.01 to 0.04)
Spinal fusion	471 (7.9)	19 854 (5.5)	0.10 (0.07 to 0.12)
CABG	155 (2.6)	4152 (1.2)	0.11 (0.08 to 0.13)
Groin hernia repair	773 (13.0)	69 806 (19.4)	0.17 (0.15 to 0.20)
Thyroidectomy	134 (2.3)	6202 (1.7)	0.04 (0.01 to 0.06)
Hysterectomy	800 (13.5)	58 177 (16.1)	0.07 (0.05 to 0.10)
Lower extremity amputation	371 (6.3)	6925 (1.9)	0.22 (0.19 to 0.25)
Appendectomy	229 (3.9)	28 348 (7.9)	0.17 (0.15 to 0.20)
Hip fracture (femoral, intertrochanteric)	45 (0.8)	5688 (1.6)	0.08 (0.05 to 0.10)
Tibial and ankle shaft fractures	168 (2.8)	16 681 (4.6)	0.09 (0.07 to 0.12)
Acuity of selected surgery at the population level			
Regularly elective	2341 (39.5)	119 364 (33.1)	0.13 (0.11 to 0.16)
Mixed elective and urgent	3148 (53.1)	190 464 (52.8)	0.01 (−0.02 to 0.03)
Regularly urgent or emergent	442 (7.5)	50 717 (14.1)	0.21 (0.19 to 0.24)
Region			
Northeast	609 (10.3)	43 047 (12.0)	0.05 (0.03 to 0.08)
Midwest	1664 (28.1)	103 300 (28.7)	0.01 (−0.01 to 0.04)
South	3077 (51.9)	162 278 (45.1)	0.14 (0.11 to 0.16)
West	575 (9.7)	51 289 (14.3)	0.14 (0.11 to 0.17)

Abbreviations: CABG, coronary artery bypass graft; COPD, chronic obstructive pulmonary disease; GERD, gastroesophageal reflux disease; GLP-1 RA, glucagon-like peptide-1 receptor agonist; SMD, standardized mean difference.

^a^ An SMD of less than 0.1 was considered indicative of good balance between groups.

**Table 2.** Unadjusted Risk of Postoperative Respiratory Complications Among Patients Who Underwent 1 of 14 Common Surgeries^a^

Complication	Patients, No. (%)		*P* value^b^
	GLP-1 RA users (n = 5931)	GLP-1 RA nonusers (n = 360 545)	
Aspiration pneumonia	42 (0.7)	2335 (0.6)	.56
Acute respiratory failure	799 (13.5)	40 060 (11.1)	<.001

Abbreviation: GLP-1 RA, glucagon-like peptide-1 receptor agonist.

^a^ Included groin hernia repair, hysterectomy, knee arthroplasty, hip arthroplasty, appendectomy, laminectomy, bariatric surgery, spinal fusion, tibial and ankle shaft fractures, colectomy, lower extremity amputation, thyroidectomy, hip fracture, and coronary artery bypass graft.

^b^ χ^2^ Test to assess differences in categorical variables in univariable analyses.

### Primary Adjusted Analysis and Sensitivity Analyses

Patients using GLP-1 RAs had no differences in odds of postoperative aspiration pneumonia (odds ratio [OR], 0.78; 95% CI, 0.57-1.06; *P* = .12) (Table 3) or acute respiratory failure (OR, 0.98; 95% CI, 0.89-1.06; *P* = .57) compared with patients not using GLP-1 RAs after adjusting for sex, age, geographic region, the aforementioned comorbidities, and surgical acuity. The results remained qualitatively consistent in the 5 sensitivity analyses (Figure).

**Table 3.** Multivariable Logistic Regression Analysis of Odds of Postoperative Aspiration Pneumonia Among Patients Who Underwent 1 of 14 Common Surgeries^a^

Characteristic	OR (95% CI)^b^	*P* value
GLP-1 RA use		
No	1 [Reference]	NA
Yes	0.78 (0.57-1.06)	.12
Sex		
Female	1 [Reference]	NA
Male	1.16 (1.07-1.26)	<.001
Age group, y		
<46	1 [Reference]	NA
46-55	1.15 (1.00-1.31)	.048
56-62	1.17 (1.02-1.34)	.03
≥63	1.94 (1.69-2.21)	<.001
Comorbidities		
Diabetes and/or obesity		
No diabetes or obesity	1 [Reference]	NA
Obesity only	0.98 (0.88-1.09)	.75
Diabetes only	1.28 (1.12-1.45)	<.001
Diabetes and obesity	1.40 (1.22-1.60)	<.001
Hypertension	1.30 (1.18-1.43)	<.001
Cancer	1.31 (1.16-1.49)	<.001
COPD	1.71 (1.49-1.95)	<.001
Chronic kidney disease	1.27 (1.09-1.49)	.003
Dementia	2.02 (1.57-2.61)	<.001
GERD	1.09 (0.97-1.23)	.13
Dysphagia	1.91 (1.45-2.51)	<.001
Gastroparesis	1.69 (0.80-3.61)	.17
Myocardial infarction	3.08 (2.64-3.59)	<.001
Heart failure	1.91 (1.65-2.21)	<.001
Peripheral vascular disease	1.74 (1.51-2.01)	<.001
Cerebrovascular disease	2.66 (2.31-3.06)	<.001
Connective tissue disease	1.08 (0.82-1.42)	.57
Peptic ulcer disease	1.34 (0.84-2.13)	.22
Liver disease	1.36 (1.14-1.61)	<.001
Hemiplegia	1.66 (1.11-2.47)	.01
AIDS	1.85 (0.99-3.47)	.06
Acuity of selected surgery at the population level		
Regularly elective	1 [Reference]	NA
Mixed elective and urgent	1.15 (1.05-1.27)	.004
Regularly urgent or emergent	1.84 (1.62-2.09)	<.001
Region		
Northeast	1 [Reference]	NA
Midwest	1.04 (0.91-1.20)	.56
South	1.08 (0.94-1.23)	.29
West	1.04 (0.87-1.23)	.69

Abbreviations: COPD, chronic obstructive pulmonary disease; GERD, gastroesophageal reflux disease; GLP-1 RA, glucagon-like peptide-1 receptor agonist; NA, not applicable; OR, odds ratio.

^a^ Included groin hernia repair, hysterectomy, knee arthroplasty, hip arthroplasty, appendectomy, laminectomy, bariatric surgery, spinal fusion, tibial and ankle shaft fractures, colectomy, lower extremity amputation, thyroidectomy, hip fracture, and coronary artery bypass graft.

^b^ Multivariable logistic regressions were used to adjust for patient demographics (age, sex, and geographic region), the comorbidities listed in the table, and surgical acuity.

**Figure.**
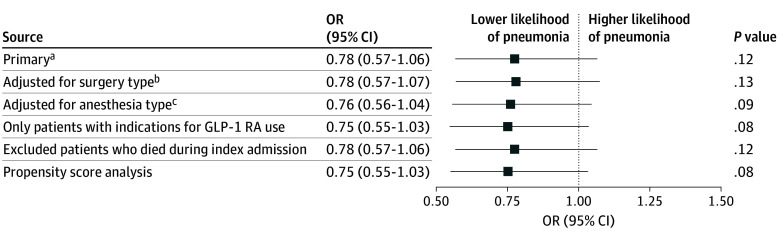
Sensitivity Analyses of Odds Ratios (ORs) for Aspiration Pneumonia Comparing Glucagon-Like Peptide-1 Receptor Agonist (GLP-1 RA) Users and Nonusers ^a^In the primary adjusted model, multivariable logistic regressions were used to adjust for patient demographics (age, sex, and geographic region), comorbidities (diabetes, obesity, hypertension, active cancer, chronic obstructive pulmonary disease, chronic kidney disease, dementia, gastroesophageal reflux disease, dysphagia, gastroparesis, myocardial infarction, heart failure, peripheral vascular disease, cerebrovascular disease, connective tissue disease, peptic ulcer disease, liver disease, hemiplegia, and AIDS), and surgical acuity. ^b^Model was re-estimated by adjusting for surgical heterogeneity with indicator variables for each specific type of surgery replacing surgical acuity. ^c^Model was re-estimated by incorporating anesthesia categories (“must be general anesthesia” vs “can be general anesthesia or other types of anesthesia”) for selected surgeries replacing surgical acuity.

## Discussion

In this cohort study, we found that preoperative GLP-1 RA use was not associated with postoperative aspiration pneumonia among patients who underwent 1 of 14 common surgeries. Similar to our findings, a recent study^17^ showed that the use of GLP-1 RAs in patients with type 2 diabetes undergoing emergency surgery was not associated with a higher risk of postoperative respiratory complications compared with no GLP-1 RA use (incidence: 3.5% vs 4.0%; OR, 0.85; 95% CI, 0.70-1.04; *P* = .12). These respiratory complications included aspiration pneumonitis and postoperative respiratory failure. Our study yielded similar results even when we expanded the study population to include both patients with and without diabetes undergoing elective, emergent, or urgent surgeries. These findings provide robust evidence in a generalized population.

Additionally, our analysis found no significant difference in the occurrence of postoperative acute respiratory failure between patients who were receiving GLP-1 RAs and those who were not, suggesting that the use of GLP-1 RAs is not associated with an increased risk of additional adverse respiratory events following surgery. Given the lack of association found between GLP-1 RA use and short-term postoperative complications, reconsidering the preoperative GLP-1 RA withholding guidelines might be beneficial.^27^ With the escalating use and broadened indications of GLP-1 RAs, health care practitioners are likely to encounter an increasing number of patients using these medications. Since there are still insufficient data to support the long-term use of GLP-1 RAs, clinicians should approach treatment of patients using GLP-1 RAs with caution. Furthermore, future studies should investigate long-term postoperative outcomes comprehensively to ensure the safe use of GLP-1 RAs.

### Strengths and Limitations

Our study has several significant strengths. First, we included the 14 most prevalent surgeries in the US, including both emergent and elective cases, to provide a broad and representative sample.^23^ This approach contrasts with previous studies, which predominantly focused on gastrointestinal procedures or emergent surgeries.^8,9,17,19^ Consequently, our findings offer greater generalizability to high-volume procedures. However, they may not be directly applicable to other surgical contexts given variations in baseline risks, anesthesia practices, and recovery processes. Second, our study used a nationwide commercial claims database, enabling a detailed exploration of a patient’s GLP-1 RA use across different practitioners and facilities. In contrast, most prior studies explored postoperative complications within a single institution.^7,18^

Our study also has limitations. First, we used GLP-1 RA prescriptions from the claims database as a surrogate for GLP-1 RA use, which limits granularity on medication adherence. Some patients with GLP-1 RA prescriptions may not take the medication before or on the day of their surgery. However, it is reasonable to assume that most patients took their GLP-1 RA before or on the day of their surgery because our study predated the 2023 ASA recommendation to discontinue GLP-1 RAs before surgery.^12^ Second, we did not include most patients with Medicare because we used a commercial claims database. However, GLP-1 RA users tend to be younger than Medicare-eligible patients, who are at higher risk for adverse effects and comorbidities.^28^ Consequently, our findings remain significant despite the exclusion of Medicare patients. Third, the categorization of surgical acuity was based on population-level characteristics, not on the individual patient-level information, due to limitation of the dataset. However, it is important to note that this population-level categorization was used in a previously published study.^23^ As such, it remains a valid and appropriate method for distinguishing surgical characteristics within this context. Fourth, the claims database lacks granularity regarding preoperative factors, such as fasting duration, gastric ultrasonography findings, or cases canceled due to aspiration concerns. This limitation may have resulted in an underestimation of aspiration events.

## Conclusions

In this cohort study, we found no significant association between GLP-1 RA use and increased short-term postoperative aspiration pneumonia. This finding suggests that it may be beneficial to reassess the preoperative withholding guidelines for GLP-1 RAs.

## Notes

Supplement 1. **eTable 1.** The 14 Common Surgeries Identified by *Current Procedural Terminology* Codes **eTable 2.** Outcome Variables Identified by the *ICD-10* **eTable 3.** Number of Patients in Each Procedure **eFigure.** Patient Selection Flowchart

Supplement 2.
Data Sharing Statement
