# Supplementary material for: Postoperative Aspiration Pneumonia Among Adults Using GLP-1 Receptor Agonists
Source: JAMA Netw Open. 2025 Mar 4;8(3):e250081. doi: 10.1001/jamanetworkopen.2025.0081 (PMC11880946; doi:10.1001/jamanetworkopen.2025.0081)
Supplement: Supplement 1. — eTable 1. The 14 Common Surgeries Identified by Current Procedural Terminology Codes eTable 2. Outcome Variables Identified by the ICD-10 eTable 3. Number of Patients in Each Procedure eFigure. Patient Selection Flowchart [file jamanetwopen-e250081-s001.pdf]

# Supplementary Online Content

Chen YH, Zink T, Chen YW, et al. Postoperative aspiration pneumonia among adults using GLP-1 receptor agonists. *JAMA Netw Open*. 2025;8(3):e250081. doi:10.1001/jamanetworkopen.2025.0081

**eTable 1.** The 14 Common Surgeries Identified by *Current Procedural Terminology* Codes

**eTable 2.** Outcome Variables Identified by the *ICD-10*

**eTable 3.** Number of Patients in Each Procedure

**eFigure.** Patient Selection Flowchart

This supplementary material has been provided by the authors to give readers additional information about their work.

© 2025 Chen YH et al. *JAMA Network Open*.

**eTable 1.** The 14 common surgeries identified by *Current Procedural Terminology* (*CPT*) codes

| **Surgeries** | **CPT code** |
|---|---|
| Bariatric surgical treatment | 43621, 43633, 43644, 43645, 43770, 43771, 43772, 43773, 43774, 43775, 43842, 43843, 43845, 43846, 43847, 43848 |
| Knee arthroplasty | 27438, 27440, 27441, 27442, 27443, 27445, 27446, 27447, 27486, 27487 |
| Hip arthroplasty | 27125, 27130, 27132, 27134, 27137, 27138 |
| Colorectal resection | 44140, 44141, 44143, 44144, 44145, 44146, 44147, 44150, 44151, 44155, 44156, 44157, 44158, 44160, 44188, 44204, 44205, 44206, 44207, 44208, 44210, 44211, 44212, 44213 |
| Laminectomy | 22220, 22222, 22224, 22856, 22857, 22861, 22862, 22867, 62287, 62351, 62380, 63001, 63003, 63005, 63011, 63012, 63015, 63016, 63017, 63020, 63030, 63035, 63040, 63042, 63045, 63046, 63047, 63048, 63050, 63051, 63055, 63056, 63064, 63075, 63077, 63081, 63082, 63085, 63086, 63087, 63088, 63090, 63091, 63101, 63102, 63103, 63170, 63172, 63173, 63180, 63182, 63185, 63190, 63191, 63194, 63195, 63196, 63197, 63198, 63199, 63200, 63250, 63251, 63252, 63265, 63266, 63267, 63268, 63270, 63271, 63272, 63273, 63275, 63276, 63277, 63278, 63280, 63281, 63282, 63283, 63285, 63286, 63287, 63290, 63709, 63740, 0202T, 0219T, 0220T, 0221T |
| Spinal fusion | 22532, 22533, 22548, 22551, 22554, 22556, 22558, 22586, 22590, 22595, 22600, 22610, 22612, 22630, 22633, 22800, 22802, 22804, 22808, 22810, 22812, 27280 |
| Coronary artery bypass graft | 33510, 33511, 33512, 33513, 33514, 33516<br>33533, 33534, 33535, 33536 |
| Groin hernia repair | 11008, 49491, 49492, 49495, 49496, 49500, 49501, 49505, 49507, 49520, 49521, 49525, 49550, 49553, 49555, 49557, 49560, 49561, 49565, 49566, 49570, 49572, 49580, 49582, 49585, 49587, 49590, 49600, 49605, 49606, 49610, 49611, 49650, 49651, 49652, 49653, 49654, 49655, 49656, 49657, 49659 |
| Thyroidectomy | 60240, 60252, 60254, 60260, 60270, 60271 |
| Hysterectomy | 58150, 58152, 58180, 58200, 58210, 58240, 58541, 58542, 58543, 58544, 58548, 58550, 58552, 58553, 58554, 58570, 58571, 58572, 58573, 58951, 58953, 58954, 58956, 59525, 58575<br>51925, 58260, 58262, 58263, 58267, 58270, 58275, 58280, 58285, 58290, 58291, 58292, 58293, 58294 |
| Lower extremity amputation | 27290, 27590, 27591, 27592, 27598, 27880, 27881, 27882, 27884, 27886, 27888, 27889, 28124, 28126, 28160, 28800, 28805, 28810, 28820, 28825 |
| Appendectomy | 44950, 44955, 44960, 44970 |
| Hip fracture surgery | 27244, 27245, 27248, 27254, 27269, 27506, 27507, 27511, 27513, 27514 |
| Tibial and ankle shaft fracture | 27535, 27536, 27540, 27758, 27759, 27766, 27769, 27784, 27792, 27814, 27822, 27823, 27826, 27827, 27828 |

© 2025 Chen YH et al. *JAMA Network Open*.

**eTable 2.** Outcome variables identified by the *International Statistical Classification of Diseases and Related Health Problems, 10th Revision (ICD-10)*

| **Primary outcome** | **ICD-10** |
|---|---|
| Aspiration pneumonia | J69 Pneumonitis due to solids and liquids<br>J69.0 Pneumonitis due to inhalation of food and vomit<br>J69.1 Pneumonitis due to inhalation of oils and essences<br>J69.8 Pneumonitis due to inhalation of other solids and liquids<br>J95.4 Chemical pneumonitis due to anesthesia<br>J95.89 Other postprocedural complications and disorders of respiratory system, not elsewhere classified |
| Other pneumonia | J13-J18<br>J13 Pneumonia due to Streptococcus pneumoniae<br>J14 Pneumonia due to Hemophilus influenzae<br>J15 Bacterial pneumonia, not elsewhere classified<br>J15.0 Pneumonia due to Klebsiella pneumoniae<br>J15.1 Pneumonia due to Pseudomonas<br>J15.2 Pneumonia due to staphylococcus<br>J15.20 Pneumonia due to staphylococcus, unspecified<br>J15.21 Pneumonia due to staphylococcus aureus<br>J15.211 Pneumonia due to Methicillin susceptible Staphylococcus aureus<br>J15.212 Pneumonia due to Methicillin resistant Staphylococcus aureus<br>J15.29 Pneumonia due to other staphylococcus<br>J15.3 Pneumonia due to streptococcus, group B<br>J15.4 Pneumonia due to other streptococci<br>J15.5 Pneumonia due to Escherichia coli<br>J15.6 Pneumonia due to other Gram-negative bacteria<br>J15.61 Pneumonia due to Acinetobacter baumannii<br>J15.69 Pneumonia due to other Gram-negative bacteria<br>J15.7 Pneumonia due to Mycoplasma pneumoniae<br>J15.8 Pneumonia due to other specified bacteria<br>J15.9 Unspecified bacterial pneumonia<br>J16 Pneumonia due to other infectious organisms, not elsewhere classified<br>J16.0 Chlamydial pneumonia<br>J16.8 Pneumonia due to other specified infectious organisms<br>J17 Pneumonia in diseases classified elsewhere<br>J18 Pneumonia, unspecified organism<br>J18.0 Bronchopneumonia, unspecified organism<br>J18.1 Lobar pneumonia, unspecified organism<br>J18.2 Hypostatic pneumonia, unspecified organism<br>J18.8 Other pneumonia, unspecified organism<br>J18.9 Pneumonia, unspecified organism |
| **Secondary outcomes** | **ICD-10** |
| Acute respiratory failure | J45, J80-84, J90, J91, J93, J95, J96, R06, R09 |

We extended our inclusion beyond aspiration pneumonia because patients with aspiration pneumonia might be coded in other ways, such as bacterial pneumonia or streptococcal pneumonia.

© 2025 Chen YH et al. *JAMA Network Open*.

**eTable 3.** Number of patients in each procedure

| Procedures | Number of patients |
|---|---|
| Total | 366,476 |
| Groin hernia repair | 70,579 (19.3%) |
| Hysterectomy | 58,977 (16.1%) |
| Knee arthroplasty | 53,841 (14.7%) |
| Hip arthroplasty | 34,118 (9.3%) |
| Appendectomy | 28,577 (7.8%) |
| Laminectomy | 25,792 (7.0%) |
| Bariatric surgery | 21,138 (5.8%) |
| Spinal fusion | 20,325 (5.5%) |
| Tibial and ankle shaft fractures | 16,849 (4.6%) |
| Colectomy | 12,608 (3.4%) |
| Lower extremity amputation | 7,296 (2.0%) |
| Thyroidectomy | 6,336 (1.7%) |
| Hip fracture (femoral, intertrochanter) | 5,733 (1.6%) |
| Coronary artery bypass graft | 4,307 (1.2%) |

© 2025 Chen YH et al. *JAMA Network Open*.

**eFigure.** Patient Selection Flowchart

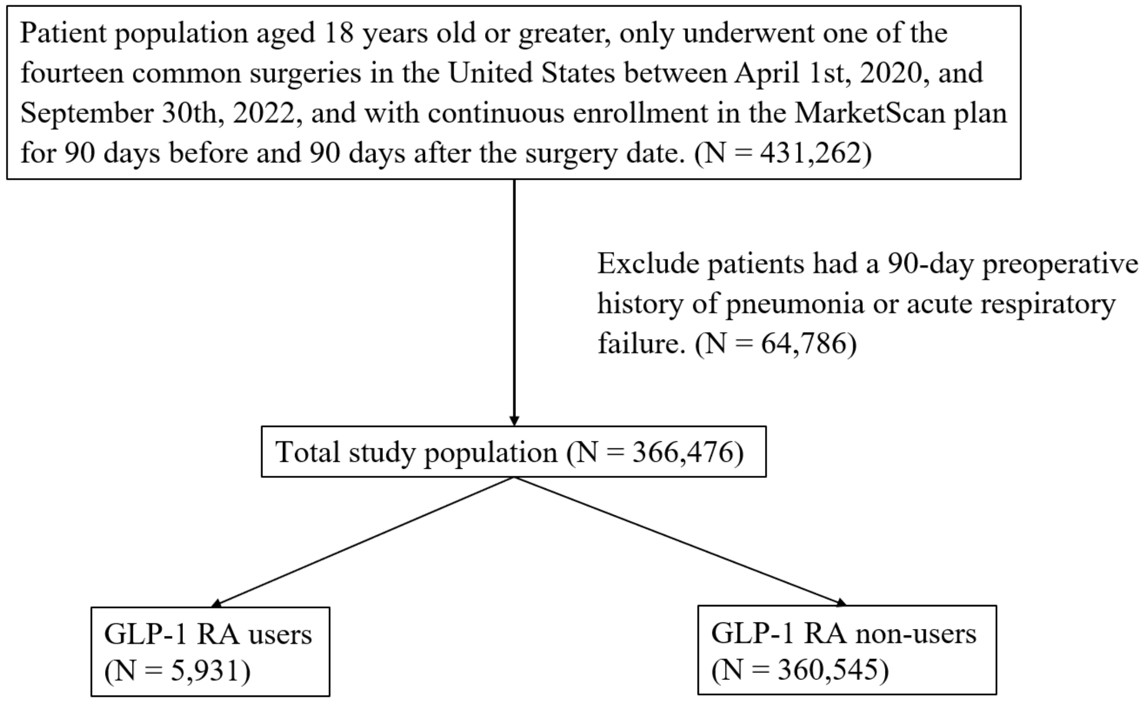


Abbreviations: glucagon-like peptide-1 receptor agonists (GLP-1 RAs).

© 2025 Chen YH et al. *JAMA Network Open*.
