# Supplementary material for: Postoperative Aspiration Pneumonia Among Adults Using GLP-1 Receptor Agonists
Source: JAMA Netw Open. 2025 Mar 4;8(3):e250081. doi: 10.1001/jamanetworkopen.2025.0081 (PMC11880946; doi:10.1001/jamanetworkopen.2025.0081)
Supplement: Supplement 2. — Data Sharing Statement [file jamanetwopen-e250081-s002.pdf]

Data Sharing Statement

Chen. Postoperative Aspiration Pneumonia Among Adults Using GLP-1 Receptor Agonists. *JAMA Netw Open*. Published March 04, 2025. doi:10.1001/jamanetworkopen.2025.0081

Data
Data available: No

Additional Information
Explanation for why data not available: The data will not be made available, as they were used under license and restrictions from Merative.
